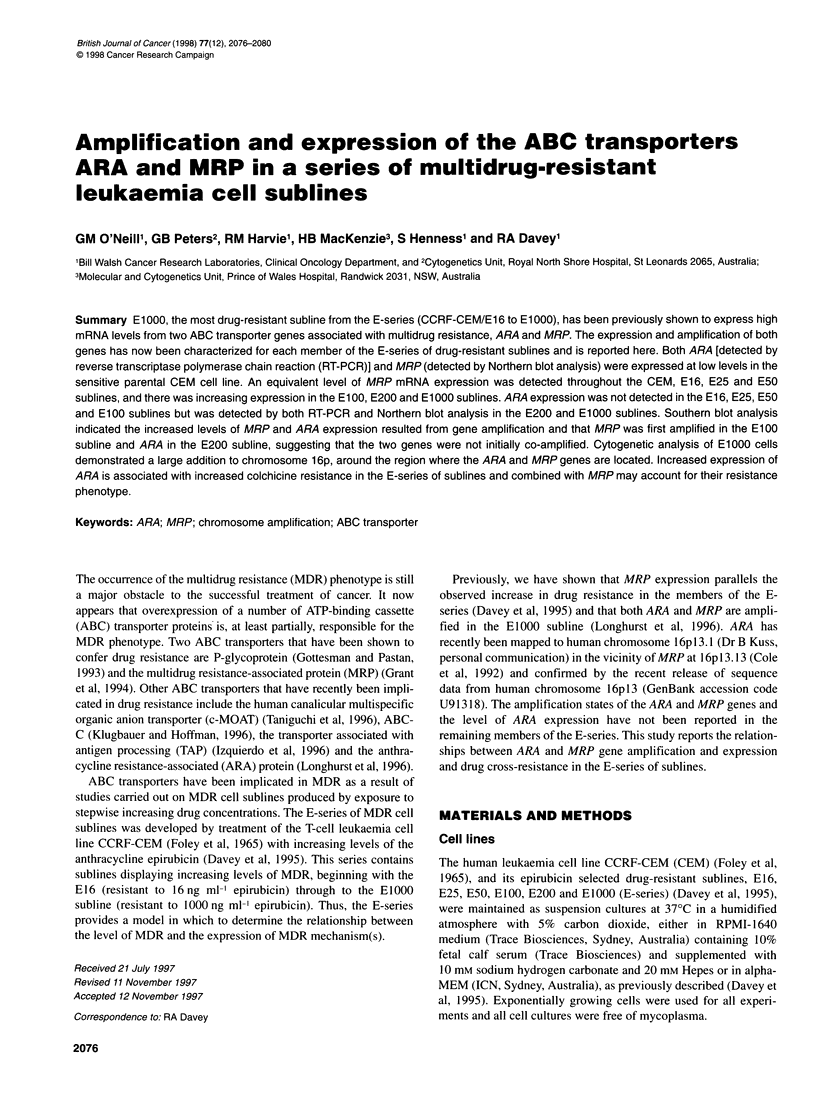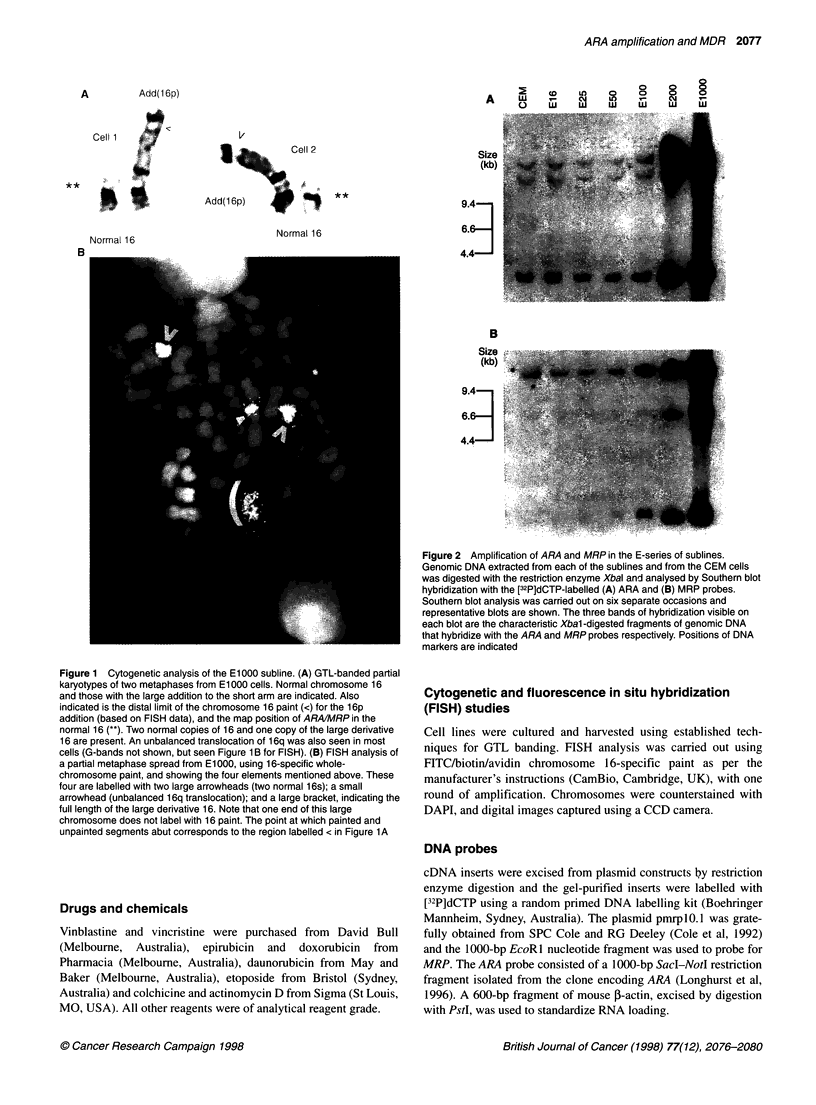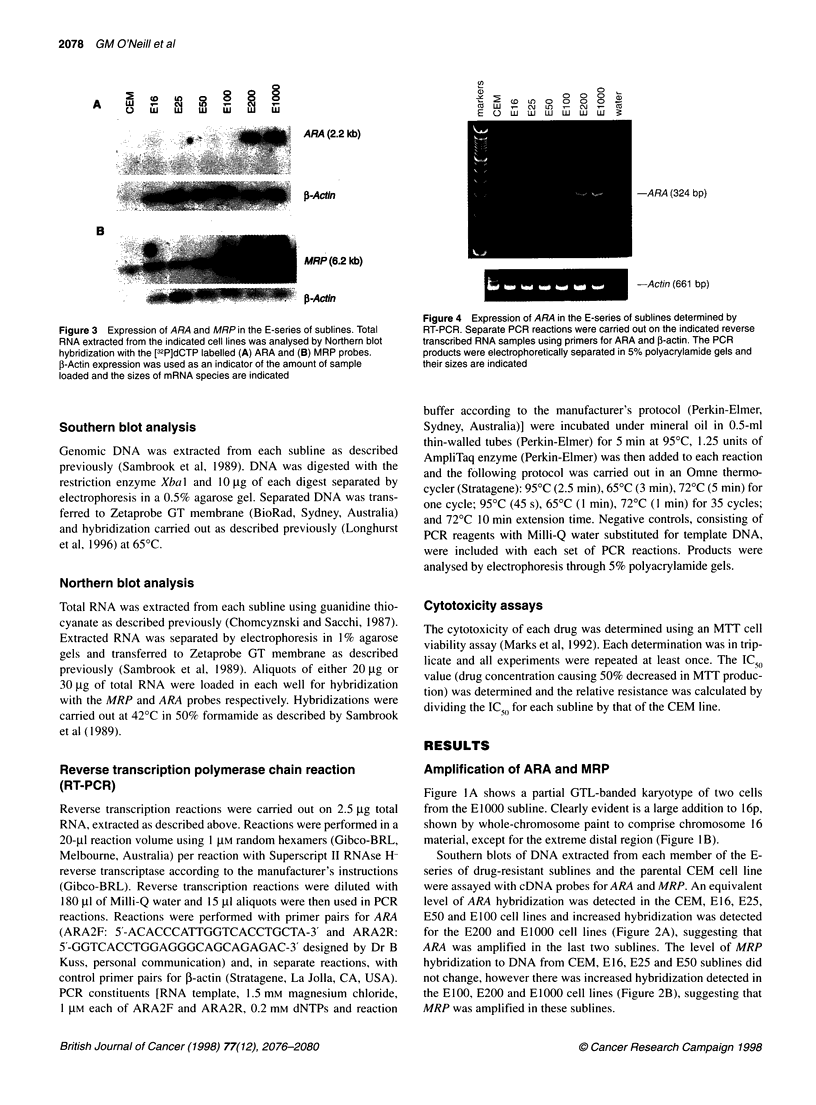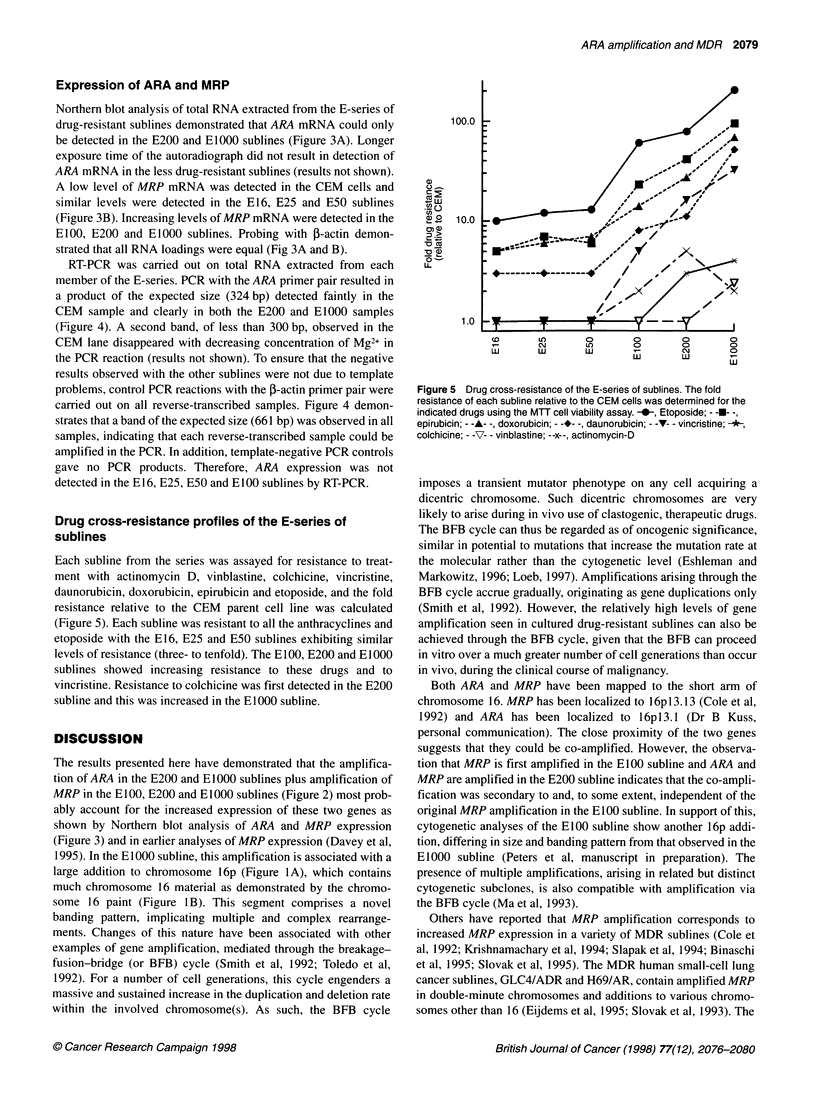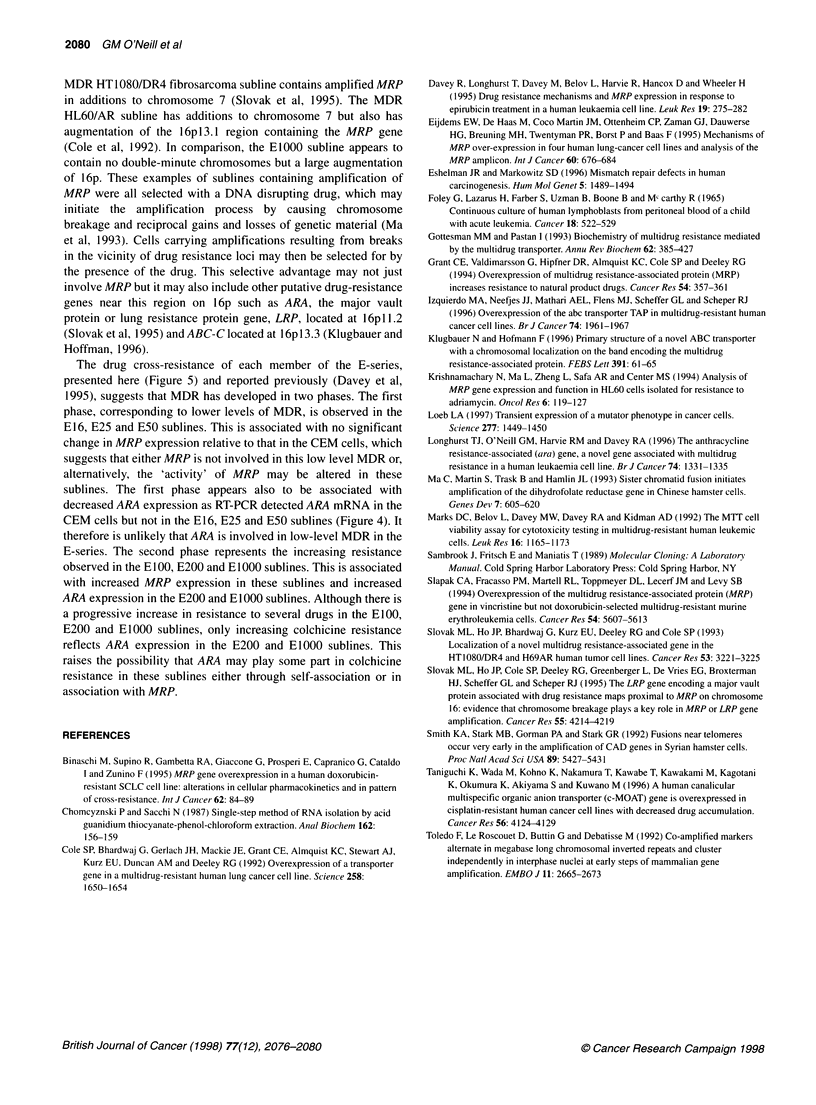# Amplification and expression of the ABC transporters ARA and MRP in a series of multidrug-resistant leukaemia cell sublines.

**DOI:** 10.1038/bjc.1998.350

**Published:** 1998-06

**Authors:** G. M. O'Neill, G. B. Peters, R. M. Harvie, H. B. MacKenzie, S. Henness, R. A. Davey

**Affiliations:** Clinical Oncology Department, Royal North Shore Hospital, St Leonards, Australia.

## Abstract

**Images:**


British Journal of Cancer (1998) 77(12), 2076–2080
© 1998 Cancer Research Campaign

### Amplification and expression of the ABC transporters ARA and MRP in a series of multidrug-resistant leukaemia cell sublines

**GM O'Neill[1], GB Peters[2], RM Harvie[1], HB MacKenzie[3], S Henness[1] and RA Davey[1]**

[1]Bill Walsh Cancer Research Laboratories, Clinical Oncology Department, and [2]Cytogenetics Unit, Royal North Shore Hospital, St Leonards 2065, Australia; [3]Molecular and Cytogenetics Unit, Prince of Wales Hospital, Randwick 2031, NSW, Australia

**Summary** E1000, the most drug-resistant subline from the E-series (CCRF-CEM/E16 to E1000), has been previously shown to express high mRNA levels from two ABC transporter genes associated with multidrug resistance, *ARA* and *MRP*. The expression and amplification of both genes has now been characterized for each member of the E-series of drug-resistant sublines and is reported here. Both *ARA* [detected by reverse transcriptase polymerase chain reaction (RT-PCR)] and *MRP* (detected by Northern blot analysis) were expressed at low levels in the sensitive parental CEM cell line. An equivalent level of *MRP* mRNA expression was detected throughout the CEM, E16, E25 and E50 sublines, and there was increasing expression in the E100, E200 and E1000 sublines. *ARA* expression was not detected in the E16, E25, E50 and E100 sublines but was detected by both RT-PCR and Northern blot analysis in the E200 and E1000 sublines. Southern blot analysis indicated the increased levels of *MRP* and *ARA* expression resulted from gene amplification and that *MRP* was first amplified in the E100 subline and *ARA* in the E200 subline, suggesting that the two genes were not initially co-amplified. Cytogenetic analysis of E1000 cells demonstrated a large addition to chromosome 16p, around the region where the *ARA* and *MRP* genes are located. Increased expression of *ARA* is associated with increased colchicine resistance in the E-series of sublines and combined with *MRP* may account for their resistance phenotype.

**Keywords:** *ARA*; *MRP*; chromosome amplification; ABC transporter

The occurrence of the multidrug resistance (MDR) phenotype is still a major obstacle to the successful treatment of cancer. It now appears that overexpression of a number of ATP-binding cassette (ABC) transporter proteins is, at least partially, responsible for the MDR phenotype. Two ABC transporters that have been shown to confer drug resistance are P-glycoprotein (Gottesman and Pastan, 1993) and the multidrug resistance-associated protein (MRP) (Grant et al, 1994). Other ABC transporters that have recently been implicated in drug resistance include the human canalicular multispecific organic anion transporter (c-MOAT) (Taniguchi et al, 1996), ABC-C (Klugbauer and Hoffman, 1996), the transporter associated with antigen processing (TAP) (Izquierdo et al, 1996) and the anthracycline resistance-associated (ARA) protein (Longhurst et al, 1996).

ABC transporters have been implicated in MDR as a result of studies carried out on MDR cell sublines produced by exposure to stepwise increasing drug concentrations. The E-series of MDR cell sublines was developed by treatment of the T-cell leukaemia cell line CCRF-CEM (Foley et al, 1965) with increasing levels of the anthracycline epirubicin (Davey et al, 1995). This series contains sublines displaying increasing levels of MDR, beginning with the E16 (resistant to 16 ng ml$^{-1}$ epirubicin) through to the E1000 subline (resistant to 1000 ng ml$^{-1}$ epirubicin). Thus, the E-series provides a model in which to determine the relationship between the level of MDR and the expression of MDR mechanism(s).

*Received 21 July 1997*
*Revised 11 November 1997*
*Accepted 12 November 1997*

*Correspondence to:* RA Davey

Previously, we have shown that *MRP* expression parallels the observed increase in drug resistance in the members of the E-series (Davey et al, 1995) and that both *ARA* and *MRP* are amplified in the E1000 subline (Longhurst et al, 1996). *ARA* has recently been mapped to human chromosome 16p13.1 (Dr B Kuss, personal communication) in the vicinity of *MRP* at 16p13.13 (Cole et al, 1992) and confirmed by the recent release of sequence data from human chromosome 16p13 (GenBank accession code U91318). The amplification states of the *ARA* and *MRP* genes and the level of *ARA* expression have not been reported in the remaining members of the E-series. This study reports the relationships between *ARA* and *MRP* gene amplification and expression and drug cross-resistance in the E-series of sublines.

### MATERIALS AND METHODS

### Cell lines

The human leukaemia cell line CCRF-CEM (CEM) (Foley et al, 1965), and its epirubicin selected drug-resistant sublines, E16, E25, E50, E100, E200 and E1000 (E-series) (Davey et al, 1995), were maintained as suspension cultures at 37°C in a humidified atmosphere with 5% carbon dioxide, either in RPMI-1640 medium (Trace Biosciences, Sydney, Australia) containing 10% fetal calf serum (Trace Biosciences) and supplemented with 10 mM sodium hydrogen carbonate and 20 mM Hepes or in alpha-MEM (ICN, Sydney, Australia), as previously described (Davey et al, 1995). Exponentially growing cells were used for all experiments and all cell cultures were free of mycoplasma.

**Figure 1** Cytogenetic analysis of the E1000 subline. (**A**) GTL-banded partial karyotypes of two metaphases from E1000 cells. Normal chromosome 16 and those with the large addition to the short arm are indicated. Also indicated is the distal limit of the chromosome 16 paint (<) for the 16p addition (based on FISH data), and the map position of *ARA/MRP* in the normal 16 (**). Two normal copies of 16 and one copy of the large derivative 16 are present. An unbalanced translocation of 16q was also seen in most cells (G-bands not shown, but seen Figure 1B for FISH). (**B**) FISH analysis of a partial metaphase spread from E1000, using 16-specific whole-chromosome paint, and showing the four elements mentioned above. These four are labelled with two large arrowheads (two normal 16s); a small arrowhead (unbalanced 16q translocation); and a large bracket, indicating the full length of the large derivative 16. Note that one end of this large chromosome does not label with 16 paint. The point at which painted and unpainted segments abut corresponds to the region labelled < in Figure 1A

**Figure 2** Amplification of *ARA* and *MRP* in the E-series of sublines. Genomic DNA extracted from each of the sublines and from the CEM cells was digested with the restriction enzyme *Xba*I and analysed by Southern blot hybridization with the [$^{32}$P]dCTP-labelled (**A**) ARA and (**B**) MRP probes. Southern blot analysis was carried out on six separate occasions and representative blots are shown. The three bands of hybridization visible on each blot are the characteristic *Xba*1-digested fragments of genomic DNA that hybridize with the *ARA* and *MRP* probes respectively. Positions of DNA markers are indicated

### Drugs and chemicals

Vinblastine and vincristine were purchased from David Bull (Melbourne, Australia), epirubicin and doxorubicin from Pharmacia (Melbourne, Australia), daunorubicin from May and Baker (Melbourne, Australia), etoposide from Bristol (Sydney, Australia) and colchicine and actinomycin D from Sigma (St Louis, MO, USA). All other reagents were of analytical reagent grade.

### Cytogenetic and fluorescence in situ hybridization (FISH) studies

Cell lines were cultured and harvested using established techniques for GTL banding. FISH analysis was carried out using FITC/biotin/avidin chromosome 16-specific paint as per the manufacturer's instructions (CamBio, Cambridge, UK), with one round of amplification. Chromosomes were counterstained with DAPI, and digital images captured using a CCD camera.

### DNA probes cDNA inserts were excised from plasmid constructs by restriction enzyme digestion and the gel-purified inserts were labelled with [$^{32}$P]dCTP using a random primed DNA labelling kit (Boehringer Mannheim, Sydney, Australia). The plasmid pmrp10.1 was gratefully obtained from SPC Cole and RG Deeley (Cole et al, 1992) and the 1000-bp *Eco*R1 nucleotide fragment was used to probe for *MRP*. The *ARA* probe consisted of a 1000-bp *Sac*I–*Not*I restriction fragment isolated from the clone encoding *ARA* (Longhurst et al, 1996). A 600-bp fragment of mouse β-actin, excised by digestion with *Pst*I, was used to standardize RNA loading.

© Cancer Research Campaign 1998 

**Figure 3** Expression of *ARA* and *MRP* in the E-series of sublines. Total RNA extracted from the indicated cell lines was analysed by Northern blot hybridization with the [$^{32}$P]dCTP labelled (**A**) ARA and (**B**) MRP probes. β-Actin expression was used as an indicator of the amount of sample loaded and the sizes of mRNA species are indicated

**Figure 4** Expression of *ARA* in the E-series of sublines determined by RT-PCR. Separate PCR reactions were carried out on the indicated reverse transcribed RNA samples using primers for ARA and β-actin. The PCR products were electrophoretically separated in 5% polyacrylamide gels and their sizes are indicated

### Southern blot analysis

Genomic DNA was extracted from each subline as described previously (Sambrook et al, 1989). DNA was digested with the restriction enzyme *Xba*I and 10 μg of each digest separated by electrophoresis in a 0.5% agarose gel. Separated DNA was transferred to Zetaprobe GT membrane (BioRad, Sydney, Australia) and hybridization carried out as described previously (Longhurst et al, 1996) at 65°C.

### Northern blot analysis

Total RNA was extracted from each subline using guanidine thiocyanate as described previously (Chomcyznski and Sacchi, 1987). Extracted RNA was separated by electrophoresis in 1% agarose gels and transferred to Zetaprobe GT membrane as described previously (Sambrook et al, 1989). Aliquots of either 20 μg or 30 μg of total RNA were loaded in each well for hybridization with the *MRP* and *ARA* probes respectively. Hybridizations were carried out at 42°C in 50% formamide as described by Sambrook et al (1989).

### Reverse transcription polymerase chain reaction (RT-PCR)

Reverse transcription reactions were carried out on 2.5 μg total RNA, extracted as described above. Reactions were performed in a 20-μl reaction volume using 1 μM random hexamers (Gibco-BRL, Melbourne, Australia) per reaction with Superscript II RNAse H$^-$ reverse transcriptase according to the manufacturer's instructions (Gibco-BRL). Reverse transcription reactions were diluted with 180 μl of Milli-Q water and 15 μl aliquots were then used in PCR reactions. Reactions were performed with primer pairs for *ARA* (ARA2F: 5′-ACACCCATTGGTCACCTGCTA-3′ and ARA2R: 5′-GGTCACCTGGAGGGCAGCAGAGAC-3′ designed by Dr B Kuss, personal communication) and, in separate reactions, with control primer pairs for β-actin (Stratagene, La Jolla, CA, USA). PCR constituents [RNA template, 1.5 mM magnesium chloride, 1 μM each of ARA2F and ARA2R, 0.2 mM dNTPs and reaction buffer according to the manufacturer's protocol (Perkin-Elmer, Sydney, Australia)] were incubated under mineral oil in 0.5-ml thin-walled tubes (Perkin-Elmer) for 5 min at 95°C, 1.25 units of AmpliTaq enzyme (Perkin-Elmer) was then added to each reaction and the following protocol was carried out in an Omne thermocycler (Stratagene): 95°C (2.5 min), 65°C (3 min), 72°C (5 min) for one cycle; 95°C (45 s), 65°C (1 min), 72°C (1 min) for 35 cycles; and 72°C 10 min extension time. Negative controls, consisting of PCR reagents with Milli-Q water substituted for template DNA, were included with each set of PCR reactions. Products were analysed by electrophoresis through 5% polyacrylamide gels.

### Cytotoxicity assays

The cytotoxicity of each drug was determined using an MTT cell viability assay (Marks et al, 1992). Each determination was in triplicate and all experiments were repeated at least once. The $IC_{50}$ value (drug concentration causing 50% decreased in MTT production) was determined and the relative resistance was calculated by dividing the $IC_{50}$ for each subline by that of the CEM line.

### RESULTS

### Amplification of ARA and MRP

Figure 1A shows a partial GTL-banded karyotype of two cells from the E1000 subline. Clearly evident is a large addition to 16p, shown by whole-chromosome paint to comprise chromosome 16 material, except for the extreme distal region (Figure 1B).

Southern blots of DNA extracted from each member of the E-series of drug-resistant sublines and the parental CEM cell line were assayed with cDNA probes for *ARA* and *MRP*. An equivalent level of *ARA* hybridization was detected in the CEM, E16, E25, E50 and E100 cell lines and increased hybridization was detected for the E200 and E1000 cell lines (Figure 2A), suggesting that *ARA* was amplified in the last two sublines. The level of *MRP* hybridization to DNA from CEM, E16, E25 and E50 sublines did not change, however there was increased hybridization detected in the E100, E200 and E1000 cell lines (Figure 2B), suggesting that *MRP* was amplified in these sublines.

© Cancer Research Campaign 1998

### Expression of ARA and MRP

Northern blot analysis of total RNA extracted from the E-series of drug-resistant sublines demonstrated that *ARA* mRNA could only be detected in the E200 and E1000 sublines (Figure 3A). Longer exposure time of the autoradiograph did not result in detection of *ARA* mRNA in the less drug-resistant sublines (results not shown). A low level of *MRP* mRNA was detected in the CEM cells and similar levels were detected in the E16, E25 and E50 sublines (Figure 3B). Increasing levels of *MRP* mRNA were detected in the E100, E200 and E1000 sublines. Probing with β-actin demonstrated that all RNA loadings were equal (Fig 3A and B).

RT-PCR was carried out on total RNA extracted from each member of the E-series. PCR with the *ARA* primer pair resulted in a product of the expected size (324 bp) detected faintly in the CEM sample and clearly in both the E200 and E1000 samples (Figure 4). A second band, of less than 300 bp, observed in the CEM lane disappeared with decreasing concentration of $Mg^{2+}$ in the PCR reaction (results not shown). To ensure that the negative results observed with the other sublines were not due to template problems, control PCR reactions with the β-actin primer pair were carried out on all reverse-transcribed samples. Figure 4 demonstrates that a band of the expected size (661 bp) was observed in all samples, indicating that each reverse-transcribed sample could be amplified in the PCR. In addition, template-negative PCR controls gave no PCR products. Therefore, *ARA* expression was not detected in the E16, E25, E50 and E100 sublines by RT-PCR.

### Drug cross-resistance profiles of the E-series of sublines

Each subline from the series was assayed for resistance to treatment with actinomycin D, vinblastine, colchicine, vincristine, daunorubicin, doxorubicin, epirubicin and etoposide, and the fold resistance relative to the CEM parent cell line was calculated (Figure 5). Each subline was resistant to all the anthracyclines and etoposide with the E16, E25 and E50 sublines exhibiting similar levels of resistance (three- to tenfold). The E100, E200 and E1000 sublines showed increasing resistance to these drugs and to vincristine. Resistance to colchicine was first detected in the E200 subline and this was increased in the E1000 subline.

### DISCUSSION

The results presented here have demonstrated that the amplification of *ARA* in the E200 and E1000 sublines plus amplification of *MRP* in the E100, E200 and E1000 sublines (Figure 2) most probably account for the increased expression of these two genes as shown by Northern blot analysis of *ARA* and *MRP* expression (Figure 3) and in earlier analyses of *MRP* expression (Davey et al, 1995). In the E1000 subline, this amplification is associated with a large addition to chromosome 16p (Figure 1A), which contains much chromosome 16 material as demonstrated by the chromosome 16 paint (Figure 1B). This segment comprises a novel banding pattern, implicating multiple and complex rearrangements. Changes of this nature have been associated with other examples of gene amplification, mediated through the breakage–fusion–bridge (or BFB) cycle (Smith et al, 1992; Toledo et al, 1992). For a number of cell generations, this cycle engenders a massive and sustained increase in the duplication and deletion rate within the involved chromosome(s). As such, the BFB cycle

**Figure 5** Drug cross-resistance of the E-series of sublines. The fold resistance of each subline relative to the CEM cells was determined for the indicated drugs using the MTT cell viability assay. –●–, Etoposide; - -■- -, epirubicin; - -▲- -, doxorubicin; - -◆- -, daunorubicin; - -▼- - vincristine; –✳–, colchicine; - -▽- - vinblastine; - -×- -, actinomycin-D

imposes a transient mutator phenotype on any cell acquiring a dicentric chromosome. Such dicentric chromosomes are very likely to arise during in vivo use of clastogenic, therapeutic drugs. The BFB cycle can thus be regarded as of oncogenic significance, similar in potential to mutations that increase the mutation rate at the molecular rather than the cytogenetic level (Eshleman and Markowitz, 1996; Loeb, 1997). Amplifications arising through the BFB cycle accrue gradually, originating as gene duplications only (Smith et al, 1992). However, the relatively high levels of gene amplification seen in cultured drug-resistant sublines can also be achieved through the BFB cycle, given that the BFB can proceed in vitro over a much greater number of cell generations than occur in vivo, during the clinical course of malignancy.

Both *ARA* and *MRP* have been mapped to the short arm of chromosome 16. *MRP* has been localized to 16p13.13 (Cole et al, 1992) and *ARA* has been localized to 16p13.1 (Dr B Kuss, personal communication). The close proximity of the two genes suggests that they could be co-amplified. However, the observation that *MRP* is first amplified in the E100 subline and *ARA* and *MRP* are amplified in the E200 subline indicates that the co-amplification was secondary to and, to some extent, independent of the original *MRP* amplification in the E100 subline. In support of this, cytogenetic analyses of the E100 subline show another 16p addition, differing in size and banding pattern from that observed in the E1000 subline (Peters et al, manuscript in preparation). The presence of multiple amplifications, arising in related but distinct cytogenetic subclones, is also compatible with amplification via the BFB cycle (Ma et al, 1993).

Others have reported that *MRP* amplification corresponds to increased *MRP* expression in a variety of MDR sublines (Cole et al, 1992; Krishnamachary et al, 1994; Slapak et al, 1994; Binaschi et al, 1995; Slovak et al, 1995). The MDR human small-cell lung cancer sublines, GLC4/ADR and H69/AR, contain amplified *MRP* in double-minute chromosomes and additions to various chromosomes other than 16 (Eijdems et al, 1995; Slovak et al, 1993). The

© Cancer Research Campaign 1998

MDR HT1080/DR4 fibrosarcoma subline contains amplified *MRP* in additions to chromosome 7 (Slovak et al, 1995). The MDR HL60/AR subline has additions to chromosome 7 but also has augmentation of the 16p13.1 region containing the *MRP* gene (Cole et al, 1992). In comparison, the E1000 subline appears to contain no double-minute chromosomes but a large augmentation of 16p. These examples of sublines containing amplification of *MRP* were all selected with a DNA disrupting drug, which may initiate the amplification process by causing chromosome breakage and reciprocal gains and losses of genetic material (Ma et al, 1993). Cells carrying amplifications resulting from breaks in the vicinity of drug resistance loci may then be selected for by the presence of the drug. This selective advantage may not just involve *MRP* but it may also include other putative drug-resistance genes near this region on 16p such as *ARA*, the major vault protein or lung resistance protein gene, *LRP*, located at 16p11.2 (Slovak et al, 1995) and *ABC-C* located at 16p13.3 (Klugbauer and Hoffman, 1996).

The drug cross-resistance of each member of the E-series, presented here (Figure 5) and reported previously (Davey et al, 1995), suggests that MDR has developed in two phases. The first phase, corresponding to lower levels of MDR, is observed in the E16, E25 and E50 sublines. This is associated with no significant change in *MRP* expression relative to that in the CEM cells, which suggests that either *MRP* is not involved in this low level MDR or, alternatively, the 'activity' of *MRP* may be altered in these sublines. The first phase appears also to be associated with decreased *ARA* expression as RT-PCR detected *ARA* mRNA in the CEM cells but not in the E16, E25 and E50 sublines (Figure 4). It therefore is unlikely that *ARA* is involved in low-level MDR in the E-series. The second phase represents the increasing resistance observed in the E100, E200 and E1000 sublines. This is associated with increased *MRP* expression in these sublines and increased *ARA* expression in the E200 and E1000 sublines. Although there is a progressive increase in resistance to several drugs in the E100, E200 and E1000 sublines, only increasing colchicine resistance reflects *ARA* expression in the E200 and E1000 sublines. This raises the possibility that *ARA* may play some part in colchicine resistance in these sublines either through self-association or in association with *MRP*.

### REFERENCES

Binaschi M, Supino R, Gambetta RA, Giaccone G, Prosperi E, Capranico G, Cataldo I and Zunino F (1995) *MRP* gene overexpression in a human doxorubicin-resistant SCLC cell line: alterations in cellular pharmacokinetics and in pattern of cross-resistance. *Int J Cancer* **62**: 84–89

Chomcyznski P and Sacchi N (1987) Single-step method of RNA isolation by acid guanidium thiocyanate-phenol-chloroform extraction. *Anal Biochem* **162**: 156–159

Cole SP, Bhardwaj G, Gerlach JH, Mackie JE, Grant CE, Almquist KC, Stewart AJ, Kurz EU, Duncan AM and Deeley RG (1992) Overexpression of a transporter gene in a multidrug-resistant human lung cancer cell line. *Science* **258**: 1650–1654

Davey R, Longhurst T, Davey M, Belov L, Harvie R, Hancox D and Wheeler H (1995) Drug resistance mechanisms and *MRP* expression in response to epirubicin treatment in a human leukaemia cell line. *Leuk Res* **19**: 275–282

Eijdems EW, De Haas M, Coco Martin JM, Ottenheim CP, Zaman GJ, Dauwerse HG, Breuning MH, Twentyman PR, Borst P and Baas F (1995) Mechanisms of *MRP* over-expression in four human lung-cancer cell lines and analysis of the *MRP* amplicon. *Int J Cancer* **60**: 676–684

Eshelman JR and Markowitz SD (1996) Mismatch repair defects in human carcinogenesis. *Hum Mol Genet* **5**: 1489–1494

Foley G, Lazarus H, Farber S, Uzman B, Boone B and M^c carthy R (1965) Continuous culture of human lymphoblasts from peritoneal blood of a child with acute leukemia. *Cancer* **18**: 522–529

Gottesman MM and Pastan I (1993) Biochemistry of multidrug resistance mediated by the multidrug transporter. *Annu Rev Biochem* **62**: 385–427

Grant CE, Valdimarsson G, Hipfner DR, Almquist KC, Cole SP and Deeley RG (1994) Overexpression of multidrug resistance-associated protein (MRP) increases resistance to natural product drugs. *Cancer Res* **54**: 357–361

Izquierdo MA, Neefjes JJ, Mathari AEL, Flens MJ, Scheffer GL and Scheper RJ (1996) Overexpression of the abc transporter TAP in multidrug-resistant human cancer cell lines. *Br J Cancer* **74**: 1961–1967

Klugbauer N and Hofmann F (1996) Primary structure of a novel ABC transporter with a chromosomal localization on the band encoding the multidrug resistance-associated protein. *FEBS Lett* **391**: 61–65

Krishnamachary N, Ma L, Zheng L, Safa AR and Center MS (1994) Analysis of *MRP* gene expression and function in HL60 cells isolated for resistance to adriamycin. *Oncol Res* **6**: 119–127

Loeb LA (1997) Transient expression of a mutator phenotype in cancer cells. *Science* **277**: 1449–1450

Longhurst TJ, O'Neill GM, Harvie RM and Davey RA (1996) The anthracycline resistance-associated (*ara*) gene, a novel gene associated with multidrug resistance in a human leukaemia cell line. *Br J Cancer* **74**: 1331–1335

Ma C, Martin S, Trask B and Hamlin JL (1993) Sister chromatid fusion initiates amplification of the dihydrofolate reductase gene in Chinese hamster cells. *Genes Dev* **7**: 605–620

Marks DC, Belov L, Davey MW, Davey RA and Kidman AD (1992) The MTT cell viability assay for cytotoxicity testing in multidrug-resistant human leukemic cells. *Leuk Res* **16**: 1165–1173

Sambrook J, Fritsch E and Maniatis T (1989) *Molecular Cloning: A Laboratory Manual*. Cold Spring Harbor Laboratory Press: Cold Spring Harbor, NY

Slapak CA, Fracasso PM, Martell RL, Toppmeyer DL, Lecerf JM and Levy SB (1994) Overexpression of the multidrug resistance-associated protein (*MRP*) gene in vincristine but not doxorubicin-selected multidrug-resistant murine erythroleukemia cells. *Cancer Res* **54**: 5607–5613

Slovak ML, Ho JP, Bhardwaj G, Kurz EU, Deeley RG and Cole SP (1993) Localization of a novel multidrug resistance-associated gene in the HT1080/DR4 and H69AR human tumor cell lines. *Cancer Res* **53**: 3221–3225

Slovak ML, Ho JP, Cole SP, Deeley RG, Greenberger L, De Vries EG, Broxterman HJ, Scheffer GL and Scheper RJ (1995) The *LRP* gene encoding a major vault protein associated with drug resistance maps proximal to *MRP* on chromosome 16: evidence that chromosome breakage plays a key role in *MRP* or *LRP* gene amplification. *Cancer Res* **55**: 4214–4219

Smith KA, Stark MB, Gorman PA and Stark GR (1992) Fusions near telomeres occur very early in the amplification of CAD genes in Syrian hamster cells. *Proc Natl Acad Sci USA* **89**: 5427–5431

Taniguchi K, Wada M, Kohno K, Nakamura T, Kawabe T, Kawakami M, Kagotani K, Okumura K, Akiyama S and Kuwano M (1996) A human canalicular multispecific organic anion transporter (c-MOAT) gene is overexpressed in cisplatin-resistant human cancer cell lines with decreased drug accumulation. *Cancer Res* **56**: 4124–4129

Toledo F, Le Roscouet D, Buttin G and Debatisse M (1992) Co-amplified markers alternate in megabase long chromosomal inverted repeats and cluster independently in interphase nuclei at early steps of mammalian gene amplification. *EMBO J* **11**: 2665–2673

 © Cancer Research Campaign 1998